# Displacement experiments provide evidence for path integration in *Drosophila*

**DOI:** 10.1242/jeb.245289

**Published:** 2023-06-16

**Authors:** Anna V. Titova, Benedikt E. Kau, Shir Tibor, Jana Mach, T. Thang Vo-Doan, Matthias Wittlinger, Andrew D. Straw

**Affiliations:** ^1^ Institute of Biology I, Faculty of Biology, Albert-Ludwigs-Universität Freiburg, 79104 Freiburg, Germany; ^2^ Bernstein Center Freiburg, Albert-Ludwigs-Universität Freiburg, 79104 Freiburg, Germany

**Keywords:** Fly, Neuroethology, Memory, Pheromones, Navigation

## Abstract

Like many other animals, insects are capable of returning to previously visited locations using path integration, which is a memory of travelled direction and distance. Recent studies suggest that *Drosophila* can also use path integration to return to a food reward. However, the existing experimental evidence for path integration in *Drosophila* has a potential confound: pheromones deposited at the site of reward might enable flies to find previously rewarding locations even without memory. Here, we show that pheromones can indeed cause naïve flies to accumulate where previous flies had been rewarded in a navigation task. Therefore, we designed an experiment to determine if flies can use path integration memory despite potential pheromonal cues by displacing the flies shortly after an optogenetic reward. We found that rewarded flies returned to the location predicted by a memory-based model. Several analyses are consistent with path integration as the mechanism by which flies returned to the reward. We conclude that although pheromones are often important in fly navigation and must be carefully controlled for in future experiments, *Drosophila* may indeed be capable of performing path integration.

## Teaser

**Highlighted Article:** Experiments that exclude the confounding effect of pheromones show that *Drosophila* remember the location of an optogenetically delivered reward.

## INTRODUCTION

Immediately after a brief taste of sugar, walking flies ‘dance’ – the walking pattern changes into looping trajectories that appear to be a local search, presumably to allow the fly to find additional sugar nearby (Dethier, 1957; White et al., 1984; Bell et al., 1985). Recently, such behaviors have been proposed to use path integration (PI) – a memory-based accumulation of distances and directions walked – to loop back to a location where yeast or sugar food was previously encountered (Kim and Dickinson, 2017; Brockmann et al., 2018; Shakeel and Brockmann, 2023 preprint). Many insect species, especially bees, ants and wasps, are known to implement path integration as a form of memory enabling a return home at the end of a foraging trip. Because flies do not have a nest, the recent work on flies used a different behavior to study path integration, namely local search for more food in the vicinity of recently encountered food (Kim and Dickinson, 2017; Brockmann et al., 2018; Shakeel and Brockmann, 2023 preprint). Although it remains unclear why a hungry fly would leave a food resource, these works propose that flies use path integration to return to the resource. Despite potential differences between flies and other species, these recent findings in *Drosophila* are exciting because they suggest that the fly neural circuit toolbox may now be used to study the neural basis of such navigation in insects. Indeed, the first steps in this direction have now been made. After optogenetically activating sugar-sensing neurons (Corfas et al., 2019; Behbahani et al., 2021; Lu et al., 2022; Haberkern et al., 2019) and dopaminergic neurons from the protocerebral anterior medial (PAM) cluster (Stern et al., 2019), flies returned to the location where they received the optogenetic activation. Thus, optogenetic activation appears capable of providing rewarding feedback similar to that of food consumption. Regardless of these apparent differences, with flies it is now possible to perform studies using computerized tracking to automatically control illumination to optogenetically reward animals at precisely defined locations and times and consequently study these behaviors using sophisticated behavioral paradigms and the genetic tools available in *Drosophila*.

Other genetic tools have been used in recent years to gain insight into the neural circuitry of underlying navigation and foraging in *Drosophila*. For example, genetically encoded calcium indicators were used to show that EPG (ellipsoid body to protocerebral bridge and gall) neurons of the central complex integrate angular information to maintain an estimate of self-heading which can be updated by locomotor turning signals, visual input or both (Seelig and Jayaraman, 2015). Recently, Lu et al. (2022) showed that silencing PFNd (columnar neurons connecting protocerebral bridge, fan-shaped body and noduli) neurons, disrupted performance in an assay designed to test path integration. Thus, it seems that as a field, we are approaching the point where we can address the behavioral and neural circuit basis for path integration in *Drosophila*.

Beyond path integration or other spatial memories, another theoretical possibility that might enable flies to return to sites of previous rewards would be the use of pheromones, which are chemical cues deposited by oneself or a conspecific. Many ant species deposit pheromone marks and use them to return to important locations. *Drosophila* are known to leave chemical deposits at sites of egg laying (Duménil et al., 2016) and nutritive sugar (Abu et al., 2018) and, at least in some circumstances, flies use such chemicals as a memory-independent cue for spatial navigation (Duménil et al., 2016; Keesey et al., 2016; Lin et al., 2015). Could past behavioral results used to support the hypothesis that flies use path integration have been influenced by a previously unknown contribution from pheromones?

Here, we show that flies deposit droplets upon being optogenetically rewarded and that naïve flies prefer such locations. The reward was delivered by stimulating sugar-sensitive Gr43a neurons with light to activate genetically encoded channelrhodopsin. Next, inspired by work on path integration in the desert ant *Cataglyphis* (Wehner and Wehner, 1986), we performed displacement experiments on flies that had been optogenetically rewarded to directly test whether *Drosophila* is capable of navigating to a remembered location after being moved away from potential pheromonal cues. Despite a potential ability of flies to use pheromones, the results of our displacement experiments are consistent with flies also being able to use path integration as a behavioral strategy to return to the reward.

## MATERIALS AND METHODS

### Fly genotypes, rearing and experimental conditions

*Drosophila melanogaster* flies used in the experiments were from a stable stock generated in the Straw lab (generated from BDSC 57636 and BDSC 55136) with genotype *+; Gr43a-Gal4; UAS-CsChrimson::mVenus* (‘*Gr43a>Chrimson*’ for short). CsChrimson is a red-sensitive channelrhodopsin (Klapoetke et al., 2014) and this genotype causes its expression in Gr43a fructose-sensing neurons (Miyamoto et al., 2012). Before experiments, flies were kept in a 25°C incubator at 60% humidity with 12 h:12 h light:dark cycles (lights on: 08:00 to 20:00 h). The flies were provided all-trans-retinal (Sigma-Aldrich R2500) within 1 day after eclosion and kept in vials wrapped into aluminium foil for 3 to 4 days. Retinal is the chromophore of CsChrimson and serves to potentiate the optogenetic response. The purpose of the foil was to prevent activation of CsChrimson- expressing neurons and degradation of retinal in the food due to light exposure. During the starvation period, flies were kept in a foil-wrapped vial with access to water but no food.

**Fig. 1.**
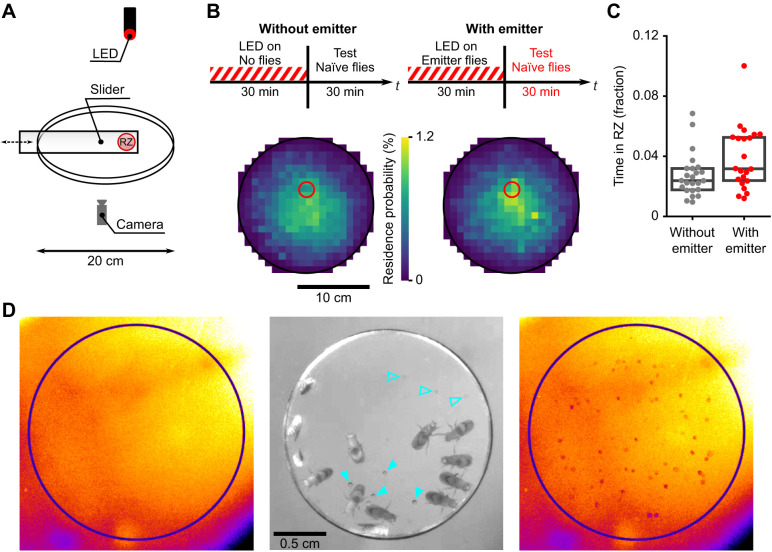
**Chemical cues deposited by optogenetically rewarded emitter flies cause accumulation of naïve flies.** (A) Experimental setup: a group of emitter flies were placed inside a slider with no floor and stood on the arena floor (RZ: reward zone). A group of naïve flies was introduced to the 20 cm diameter arena after the emitter flies were removed via the slider. (B) Top: timeline of the experiment in which emitter flies were illuminated with flickering LED light to activate Gr43a sugar-sensing neurons expressing excitatory optogenetic channels. Bottom: frequency of naïve test flies present in a location (residence probability) during the test stage for two conditions: without emitter flies (left, *n*=24 groups) or with emitter flies (right, *n*=22 groups). (C) Fraction of time spent in RZ by naïve flies during the test stage with and without emitter (*P*=0.04, two-tailed Welch's *t*-test for unpaired samples). Every dot represents fraction of time spent in the RZ by a group of naïve flies (group size: 10–15 individuals). Boxes are from first to third quartile with median indicated as a horizontal line. (D) Close-up view of the small reward zone chamber portion of the arena during different stages. Left: arena surface prior to fly introduction (false color, see Materials and Methods); middle: emitter flies in the slider chamber; right: arena floor after emitter flies were removed (false color, same color scale as left panel). The pointers indicate the fresh droplets (solid) and the dried-out droplets (transparent). The images were taken with the tracking IR camera and processed to increase visibility of the droplets (see Materials and Methods).

For the pheromone experiment, we used mixed groups of 4- to 6-day-old male and female *Gr43a>Chrimson* flies. One day before the experiment, the flies were briefly anesthetized at 4°C and put to starve together for at least 24 h. For the experiment, the flies were transferred directly to the arena using a pipette tip with a tube. The images in Fig. 1D were obtained in an independent trial performed on a group of female flies.

For the displacement experiment, we used 4- to 5-day-old female flies, starved for 24–30 h. One day before the experiment, the flies were briefly anesthetized at 4°C, sorted by gender and put to starve. Approximately 1 h before start of the experiment, flies were moved into small individual containers (pipette tips) under dim light and placed in a dark plastic box. At the start of the experiment, the fly was taken from the box and placed in the arena. To transfer the flies, we applied airflow through a tube attached to the pipette tip. Displacement experiments were conducted in the late afternoon (15:00–20:00 h). The temperature in the room was maintained at ∼20°C.

### Optogenetic rewards using tracking and stimulation software

Real-time tracking was based on background subtraction (Strand Camera, https://strawlab.org/strand-cam) using a machine vision camera and lens (Basler Ace acA1300-200um and Tamron 12VM412ASIR), near infrared illumination (WINGER WEPIR1-S1 IR Power LED Star 850 nm 1 W) and filter (Lee Infrared Transmission 87 Filter). Flies were rewarded by optogenetic activation of sugar-sensitive neurons (e.g. Corfas et al., 2019). In this technique, we used neurogenetic tools to drive genetic expression of a red-light-sensitive cation channel (CsChrimson) in sugar-sensitive (Gr43a-expressing) cells. Thus, by illuminating them with red light, flies should experience the rewarding taste of sugar (Corfas et al., 2019). To deliver the optogenetic stimulus, we used a software controlled red light-emitting diode (WINGER WEPIR1-S1 Power LED Star Red 625 nm 1 W). In the pheromone experiment, stimulation was carried out periodically using a 1 s-on/2 s-off toggling mechanism, independently of the flies' positions. In the displacement experiment, the LED was turned on if and only if the fly was inside the experimenter-defined and computer-controlled reward zone (RZ) during the stimulation period. The stages of the experiments were controlled automatically by a Python script that interacted with the tracking software. The tracking software produces trajectory recording files in csv format consisting of the coordinates of detected objects and the state of the stimulation LED at each frame. These recorded trajectories are referred to as ‘raw recordings’.

All data analysis scripts, image processing macros and pheromone model are available from Dryad (Straw et al., 2023) at: https://doi.org/10.5061/dryad.vdncjsz0b and GitHub at: github.com/strawlab/titova_et_al_displacement_supplemental. All figures in this manuscript, including from the experiments described below, were created using the FigureFirst package (Lindsay et al., 2017).

### Pheromone experiment

#### Apparatus

For the pheromone experiment, three identical setups were used in parallel. Each experimental setup consisted of a light-tight box with infrared LED illumination, IR-sensitive camera for tracking flies in darkness, a red LED for stimulation and a 20 cm diameter walking arena (Fig. 1A).

The walking arena had circular glass floor and a 3 mm high wall. To prevent the flies from going to the edge of the arena, the wall was a 20 cm diameter aluminum ring heated by a Copper Manganese Nickel alloy heat resistance wire (ISOTAN, 2.5 Ohm m^−1^, 0.5 mm diameter) glued with thermally conductive adhesive. A transparent acrylic lid coated with a slippery, transparent layer (Sigmacote, Sigma-Aldrich) prevented the flies from leaving or walking on the lid.

The ‘emitter flies’ (terminology from Abu et al., 2018), which served to putatively emit pheromones, were introduced to the arena contained in a circular chamber within the slider (diameter of the chamber: 20 mm, height of the slider: 3 mm, as the arena). The floor and ceiling of the chamber in the slider were formed by the floor and ceiling of the arena. At the start of the experiment, the slider was moved into the arena so that the chamber was located at the optogenetic reward zone, thus allowing the emitter flies to walk on the arena floor only inside this zone. After the optogenetic activation of the emitter flies, the slider was removed through the hole in the arena wall, without opening the arena lid, and then the naïve test flies were introduced to the arena. During the slider movement, a thin sheet was placed under the slider and above the arena floor to prevent deposits outside of the reward zone. Between trials, the floor was wiped with ethanol.

#### Experimental design

The experiment consisted of two stages: a stage with emitter and a stage with test naïve flies (Fig. 1B). During the first stage, the slider was inside the arena and the stimulation LED was periodically toggled (1 s on/2 s off). There were two experimental conditions: with emitter – a group of emitter flies (10–15 flies in a group) was inside the small slider chamber during the first stage; without emitter – no flies were in the slider chamber. After 30 min, the slider was removed, naïve flies (a group of 10–15 flies) were introduced into the arena (using pipette tips with a cut opening to transfer the flies) and their locations were recorded for another 30 min. Data collection was stopped at the end of an experimental day when *n*=20 trials was reached for both experimental conditions, resulting in *n*=22 trials with emitter and *n*=24 without emitter flies. We did not perform power analysis to determine sample size.

#### Data analysis

The position data for naïve flies from the test stage of the pheromone experiment were downsampled by time to 10 Hz before further analysis. Upon being introduced to the arena, the flies were not active and spent a lot of time stopped in apparently random locations. Therefore, we excluded first 5 min of the test stage from the analysis shown in Fig. 1B,C. An alternate analysis in which the initial 5 min of the test period were included showed qualitatively similar results (data not shown).

To compare locations in which the flies spend most of the time, we plotted residency histograms, which are heatmaps showing the frequency of being present in a location, measured as the relative amount of trajectory points falling into square-shaped bins of the arena floor (Fig. 1B, bin size 1 cm×1 cm). The resulting heatmap for the experimental condition was created by averaging the heatmaps of individual trials, where each trial corresponded to a single group of flies tested. An alternate analysis in which stopped periods were excluded and consequently only walking was considered, as performed for the displacement experiment analysis, resulted in qualitatively similar results (not shown).

To characterize the amount of time flies spent in the reward zone we computed fraction of trajectory points falling into the area of interest for each recording of the test stage. We compared the distributions of these fractions between conditions (Fig. 1C). For statistical comparison we used Welch's *t*-test for samples with unequal variances.

The images in Fig. 1D were obtained as screenshots from the IR tracking camera, cropped to show the reward zone. The circles on the left and right panels of Fig. 1D indicate the reward zone, the central image shows the slider chamber with the emitter flies in it. To increase the visibility of the released droplets, the images were processed in ImageJ. For the image with flies present, we applied the ‘adjust color balance’ tool with settings min: 0, max: 91. The images without flies were converted to 8-bit images with lookup map ‘Fire’ and then brightness/contrast adjustment (settings: min, 160; max, 245) was applied.

### Pheromone model of local search in a circular channel

A model of pheromone-mediated behavior was inspired by Behbahani et al. (2021) and some of the parameters were reproduced from their work. The model consists of a circular linear channel with reward zones and a fly agent that can perform discrete steps (time step, 0.5 s) in the channel in two directions and release a pheromone droplet at any step. The agent is initiated in global search mode, where it continuously walks forward in one direction. When the agent locates the reward (activation zone), it switches to the local search mode. A reward zone can be in one of two states: activated or deactivated. When the agent steps on an activated reward, it eats (does not move for 10 time steps), releases a pheromone, chooses a new run length *r*_rew_ and continues walking in the same direction for distance *r*_rew_ before making a reversal turn. Every time the agent encounters the activated reward, the reward deactivates for a refractory period of 16 time steps. If the agent steps on a released pheromone, it chooses a new run length *r*_ph_ which depends on the current odor value of that droplet. When the agent finishes a run, it performs a reversal and chooses a new run length *r*_0_ sampled from a wide baseline distribution. The odor value of a pheromone decays over time linearly from 1 to 0 over time *τ*:
(1)

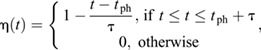


where *t*_ph_ is the moment of pheromone droplet release. The current run length of an agent is defined by the last action it performed: eating, smelling or reversal, and does not rely on spatial memory. The run length is chosen from a normal distribution with corresponding parameters.

Reversal:
(2)

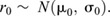
Eating (reward):
(3)

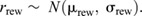
Smelling:
(4)

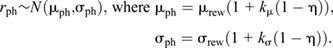


Fig. S1 was generated with the following set of parameters: μ_0_=80; σ_0_=20; μ_rew_=4.125; σ_rew_=2.625; *k*_μ_=3; *k*_σ_=3; τ=500. The parameters for ‘eating’ were taken from Behbahani et al. (2021), FR model. Two experiments from the Behbahani study were modeled here: the ‘circling’ experiment and the ‘multiple rewards’ experiment.

In the circling experiment model, 6 trials in a row were performed on each fly in a channel with a 26 body length (BL) circumference and the activation zone position is switched between the opposite sides of the channel on every trial. Each trial has a 5 min activation period (AP) and a 5 min post-activation (post-AP) period.

The modeling for the multiple rewards experiment was performed in a bigger 52 BL circumference channel with 3 rewards close to each other (coordinates: 0, ±5 BL), one trial with 5 min AP and 5 min post-AP periods for each simulation. The run midpoint was defined as the middle between two consecutive reversals, as in Behbahani et al. (2021). Our model is available from Dryad, Zenodo and Github (see Data availability).

### Displacement experiment

#### Apparatus

**Fig. 2.**
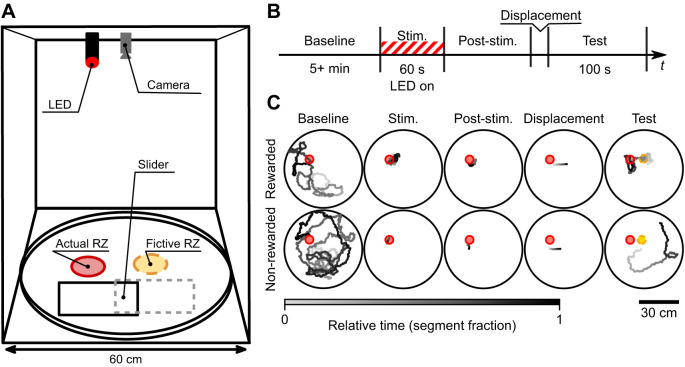
**Displacement experiment for freely walking flies tests whether flies use path integration in preference to pheromonal cues.** (A) Experimental setup. In the 60 cm arena, when a fly leaves the reward location after the stimulation period and eventually stands on a thin slider, it is manually displaced. (B) Timeline of the displacement experiment for rewarded flies. During the baseline period (‘Baseline’, 5–25 min), the fly is introduced to the arena and the reward zone (RZ) is activated after 5 min. The stimulation period (‘Stim.’, 60 s red light on) is started when the fly finds the activated RZ, and the red light is on if and only if the fly is in the RZ. The RZ is deactivated when the fly leaves the RZ for the first time after the LED on time exceeds 60 s. The post-stimulation period (‘Post-stim.’, 3–100 s) lasts until the fly walks onto the slider. In the displacement period (about 3 s), the experimenter manually pulls the slider via nylon line with the fly on it. The test period is the first 100 s after displacement. For control experiments with non-rewarded flies, the timeline was similar, but the stimulation was not performed. (C) Examples of recorded trajectories during different experimental stages. Top row, rewarded fly; bottom row, non-rewarded fly. More examples are presented in Fig. S2C.

The flies walked freely in a circular arena with 60 cm diameter and 2 cm high walls, glass floor and transparent acrylic lid (Fig. 2A).

The arena border was made of a bicycle wheel rim, with heat wire (described above) thermally glued to the outer side, to create a heat barrier preventing flies from going to the edge and climbing the walls. To regulate temperature in the arena, small gaps were created by putting washers between the arena floor and the walls, as well as between the walls and the lid. The temperature profile is plotted in Fig. S2A.

The entire arena, camera, lens and IR LED illumination (generic low power 890 nm LEDs inserted into the spoke holes) were located inside a light-tight cardboard box with black walls and ceiling and white ground.

To optogenetically stimulate the *Gr43a>Chrimson* flies, the red LED was focused to illuminate a circular part of the arena floor. The LED stimulation was automatically performed only during the stimulation period (see ‘Experimental design’ below) when the fly was inside the illuminated area (actual RZ), namely a circle with center (*x*=−9.8 cm, *y*=6.7 cm relative to the arena center) and 2.8 cm radius. The measured values of the stimulation light intensity are in Fig. S2B. As discussed in the introduction, the optogenetic activation of Gr43a sugar neurons is expected to be an appetitive reward for the flies.

The displacement of the fly was performed using a transparent plastic sheet (A4). It was placed on the arena floor so that the border of the sheet was about 1 cm from the RZ. Two nylon monofilament lines were taped to the side of the sheet, leading outside of the light-tight box where they were pulled by the experimenter to displace the fly. Two lines were used to pull two corners of the slider without inducing rotational motion.

#### Experimental design

The experimental timeline is illustrated in Fig. 2B. The experiment consisted of several stages performed in series controlled automatically by a custom Python script. The initial stage was a 5 min baseline period during which no optogenetic stimulation was performed. This baseline period allowed the fly to explore the arena in darkness. Starting from minute 5, the reward zone (RZ) was active – the presence of the fly would trigger illumination by the red LED. This commenced the onset of the stimulation stage. During this stimulation period, red LED was automatically turned on when the fly entered the RZ. When the fly exited the RZ, the light automatically turned off. The LED on time was accumulated and when it reached the limit (60 s for rewarded flies, see below for non-rewarded flies), the stimulation period ended. After this stimulation period, when the fly exited the RZ, the red light turned off and would not turn on again. This protocol ensured that all subjects received a minimum cumulative amount of stimulation, with total duration of the stimulation stage being variable across trials. Once the fly walked on the plastic sheet slider after stimulation, the slider with the fly on it was displaced by manual pulling on the attached monofilament lines. The experimenter attempted to pull smoothly and minimize jerk. Because the pull was performed manually, the distance, speed and acceleration were unfortunately variable across trials (mean length±s.d.: 92±27.8 mm). Therefore, the fictive RZ location was trial specific (Fig. S3B). The recording was stopped 10 min after the end of stimulation. For analysis, we used the first 100 s after displacement as the test period. We use the term ‘actual RZ’ to describe the reward zone at which the flies received optogenetic stimulation, and may have left pheromonal deposits. This is distinct from the fictive RZ, whose location is calculated as the location of the actual RZ shifted by the distance and direction of displacement.

As a control, a similar experiment was performed on flies of the same genotype (*Gr43a>Chrimson*), but with no reward. All procedures were identical to that described above with two changes. First, the LED was never turned on and thus no optogenetic reward was provided. Second, owing to flies no longer being attracted to the so-called reward zone, we shortened the so-called stimulus period to 0.5 s so that the next experimental period could be reached. All other aspects of the experiment were performed identically between the rewarded and non-rewarded control conditions.

Data collection was stopped when *n*=20 successfully finished trials was reached for both conditions. We did not perform power analysis to determine sample size. We aborted the trial in several conditions: (1) if the fly did not find the RZ in 20 min after the RZ activation; (2) if during the stimulation period the fly did not return to the RZ for more than 2 min; (3) if the fly started flying during displacement. In the rewarded condition, 19 out of 65 trials finished successfully, in the non-rewarded condition, 20 out of 42 were successful. One trial in the rewarded condition was discarded at the analysis stage because of hardware problems during the recording.

Between the trials, the arena floor and the slider surface were cleaned with 70% ethanol.

#### Data analysis

Before the displacement experiment analysis, falsely detected points (e.g. outliers from reflections of the LED light being detected by the fly tracking software) were removed from the recordings of flies' coordinates. These are typically obvious artifacts validated by comparing the tracking data with video files recorded simultaneously. For example, during displacement, moving the pulling lines reflected IR light and caused such points in the recording. The displacement period was identified by manual selection of the corresponding trajectory segment (a relatively straight movement shortly after end of stimulation).

**Fig. 3.**
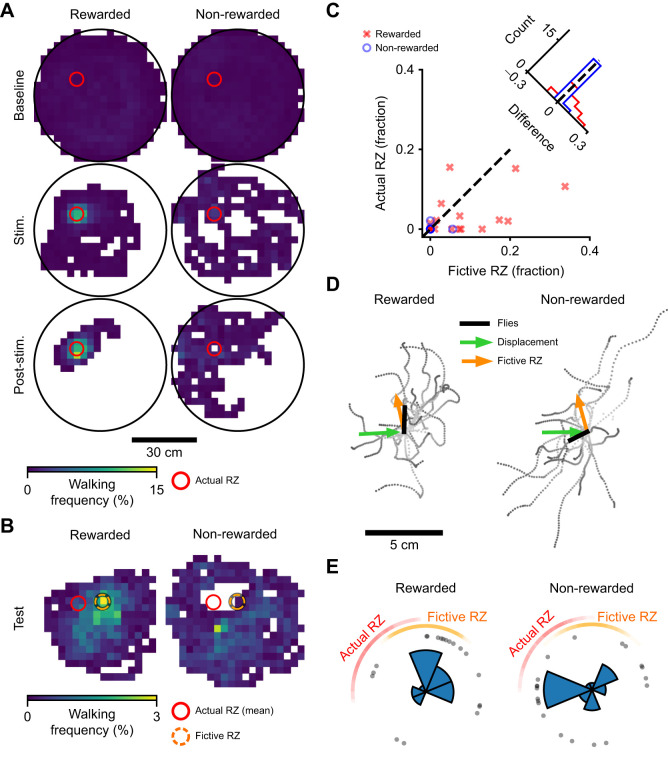
**Fly behavior after displacement is consistent with path integration.** (A) Walking frequency histograms before displacement for rewarded (*n*=19) and non-rewarded (*n*=20) flies: baseline period, light off; stimulation period (Stim.), light on when the fly is inside the reward zone (RZ); post-stimulation period (Post-stim.), light off. (B) Walking frequency histograms during the first 100 s after displacement (test period) in the fictive reward coordinate system (each individual fly trajectory was aligned to the fictive RZ, orange dashed circle, before making the histogram). The red circle represents the mean position of actual RZ. (C) Fraction of time spent by the flies inside actual and fictive reward zones during the test period (scatter plot) and histograms of corresponding fraction differences. Two-tailed paired *t*-test: rewarded, *P*=0.029; non-rewarded, *P*=0.572. (D) Trajectories of the flies during the first 5 s after displacement, shifted to the release point, with indicated trajectory vector average (‘flies’), mean direction of displacement and mean direction to the fictive reward zone. (E) Walking directions in the first 5 s after displacement. Each dot represents one fly. Data are rotated such that vertically upwards indicates direction to fictive RZ. We exclude the possibility that the rewarded flies walked in uniform directions (Rayleigh test for uniformity: *P*=0.018, *z*=3.894), whereas non-rewarded flies showed no difference from uniformity (*P*=0.352, *z*=1.057). Central circular histograms show all flies. The arcs show the range of directions towards the actual (red) and fictive (orange) reward zones.

To see whether the flies tend to perform local search, and where it occurs, we calculated walking frequency histograms for each stage of the experiment (Fig. 3A,B; Fig. S3C). These specifically exclude periods in which the flies are stopped. We describe two aspects in detail here. First, because the manual pulling caused a different displacement for each individual fly, to analyze the statistics of fly locomotion relative to the fictive reward zone, we plotted the post-displacement trajectories in a coordinate system relative to the fictive RZ (Fig. 3B). The fly position in the fictive RZ coordinate system is defined as the position in the original coordinate system translated by the distance and direction of the displacement. The same data are plotted in the original, arena coordinate system in Fig. S3C. The second aspect about the walking frequency histograms we describe in detail here is the reason and method for including walking, but not stopping in these histograms. The reason is that flies tend to stop in apparently random locations for long periods of time and these stops result in large peaks on the histograms, obscuring the local search trajectories during movement (Fig. S3A, left column). The method for creating these walking frequency histograms is as follows. A two-stage downsampling was performed. First, the trajectories were downsampled to 0.1 s time intervals to reduce high-frequency, low-amplitude tracking noise and number of analyzed points (Fig. S3A, middle). Next, the trajectories were downsampled by cumulative distance (Fig. S3A, right), with steps of 1 mm. Downsampling by distance removes the stops from the data. The resulting heatmaps were constructed from downsampled trajectories pooled for experimental condition and show the relative number of points falling into square-shaped bins (bin size 1 cm×1 cm).

The fraction of time spent by the fly in the actual or fictive reward zone after displacement (Fig. 3C) was calculated based on the trajectories downsampled by time with step size of 0.1 s. We used a two-tailed paired *t*-test to compare the fractions spent in fictive versus actual RZ by individual flies. The distance to reward zone was defined as the distance from the fly to the center of the reward zone. The distribution of distances to actual and fictive reward zones (Fig. S3D) was plotted based on the two stage downsampled trajectories (described above).

To analyze the directions of walking after displacement, we plotted fly trajectories during the first 5 s after displacement (Fig. 3D). The axes were translated to the release point (end of displacement) and the walking directions from this point were compared in different conditions. We calculated the mean fly trajectory vector (black line in Fig. 3D) as the vector sum of individual fly locations after walking in these 5 s. The mean direction of displacement and mean direction to fictive reward zone (green and orange arrows, respectively, in Fig. 3D) were calculated from angles and disregarded the distances. For each fly, we calculated the direction of movement in first 5 s relative to the fictive reward zone (Fig. 3E) and performed a Rayleigh test for unimodal deviations of circular uniformity independently for rewarded and non-rewarded conditions, using the test.rayleigh function from Python package pycircstat (https://github.com/circstat/pycircstat/). The mean direction was calculated as angular component of vector average of all directions represented as unit vectors.

To check if the flies retrace their steps using a potential chemical trail on the slider surface, we evaluated the amount of overlap between the trajectory just before displacement and just after displacement, taking all data within 2 cm of the fly's location at displacement (Fig. S3F,G). A point of the ‘after’ trajectory was labeled as overlapping if there existed a point of the ‘before’ trajectory located less than 2.5 mm away. The intersection score was calculated as the maximal distance between the ends of fully overlapping trajectory segments. See intersection_score.ipynb file (https://github.com/strawlab/titova_et_al_displacement_supplemental) for the script used to calculate these values.

## RESULTS

### Visitation of particular spatial locations can be mediated by pheromones

To address the potential role of pheromones in a fly navigation task, we checked if the previous presence of optogenetically rewarded flies could affect the spatial behavior of naïve flies introduced to the arena later (Fig. 1). The first group of flies, *Gr43>CsChrimson* flies exposed to optogenetic activation light, are here called emitter flies to suggest that they may putatively emit pheromones (terminology of Abu et al., 2018). We contained a group of emitter flies in a smaller area inside the arena, the reward zone, by using a slider with a circular chamber, exposed them to optogenetic reward and removed the slider with the flies from the arena before introducing a group of naïve test flies. We compared the behavior of naïve flies in two conditions: a condition with emitter flies and a control condition without emitter flies which was otherwise treated identically. During the initial 30 min stage, with or without a group of emitter flies, a flickering red LED illuminated the reward zone (RZ). Gr43a sugar taste neurons in emitter flies were activated by the optogenetic stimulation, which presumably made the flies experience the rewarding taste of sugar. After this initial reward stage, the slider was removed from the arena which, in the condition with emitter flies, also removed them from the arena (Fig. 1A). Naïve flies were introduced into the dark arena and their position tracked for 30 min using infrared illumination and camera. Histograms of naïve fly positions showed greater residence levels close to the reward zone for the trials in which emitter flies had previously been optogenetically rewarded (Fig. 1B). The fractions of time each test group of flies spent inside the reward zone (Fig. 1C) were statistically significantly different between the two conditions (*P*=0.04, unpaired two-tailed Welch's *t*-test; *N*_emitter_=22 groups, *N*_no_emitter_=24 groups). Photographs before, during and after emitter flies were rewarded (Fig. 1D, Movie 1) show droplets deposited by the flies. While we did not perform chemical analysis on these droplets, they, or other invisible chemical cues, were sufficient to cause naïve flies to accumulate at the site of reward and could be used as a navigational strategy available to *Drosophila* when performing optogenetic reward experiments.

These results do not allow us to distinguish whether accumulation of naïve flies depends on the emitter flies having been rewarded, as we tested only rewarded flies. Regardless, these results highlight the possibility that chemical cues deposited by emitter flies, even in a potentially reward-independent way, could cause accumulation of naïve flies. Previous studies which used fly accumulation as evidence for path integration generally have not controlled for the potential effect of fly-emitted pheromones (Corfas et al., 2019; Stern et al., 2019) and therefore we argue that they cannot be used as strong evidence that flies are indeed capable of idiothetic path integration. As an exception, one study made use of flies in which oenocytes, cells which produce cuticular hydrocarbons and pheromones, were genetically ablated (Kim and Dickinson, 2017). However, we note in those experiments that traces of yeast odor may have remained at or near the reward location. We argue that this may have facilitated local search near the reward location owing to chemical cues, not necessarily path integration. Other experiments by Kim and Dickinson (2017) used a slider to remove food after a fly visit, but we suggest that food odor in the air, liquid food spread from the slider during walking or pheromones deposited near but not on the slider could again have been used to mediate a local search centered at the food location.

One line of evidence used to argue that flies perform path integration comes from recent experiments in which flies were given an optogenetic reward, again by activation of sugar-sensing neurons, in a specific location along a circular channel (Behbahani et al., 2021). In that work, repeated visits to the rewarded location, especially after multiple cycles of walking around the circular channel, were interpreted as evidence of path integration. To address whether these results might be theoretically explained by a pheromone-based mechanism rather than a path-integration mechanism, we created an agent-based model performing the same task (Fig. S1). In our model, a simulated fly agent can emit pheromones that decay in intensity over time but this agent has no ability to remember the locations of previous rewards. The statistics of the agent behavior in this model were similar to those observed in real flies and we tested simulated behavior in two experimental designs based on those of Behbahani et al. (2021). In the first design, reward location alternated between two opposite sides of the circular channel, and we observed a distribution of ‘run midpoints’ (see Materials and Methods for definition) similar to that measured experimentally (Fig. S1A–C). As their experimental design alternated rewarded sides, Behbahani and colleagues argued that lack of 180 deg periodicity in the data is inconsistent with pheromone usage. In the pheromone-based model, lack of 180 deg periodicity is due to the pheromone signal decaying within the 10 min alternations used in the experiments. The second experimental design used the same model with three reward locations, and we obtained a distribution of run midpoints that was similar to those both of the path integration model of Behbahani et al. (2021) and their experimental results (Fig. S1D–F). In these simulations, we designed the model structure and manually adjusted parameters to check if a pheromone-based agent model could approximate the behavioral data and found that indeed it could. While a closer match to behavioral data could be made by fitting experimental data with optimization algorithms, this is not necessary to establish the fundamental point that a pheromone-based mechanism would be sufficient to reproduce several lines of evidence used to argue that flies are capable of path integration.

Our experiments (Fig. 1), those of others (Duménil et al., 2016; Lin et al., 2015) and our modeling (Fig. S1) show that pheromones need to be considered as a potential mechanism by which flies return to rewarding locations in the environment. Furthermore, experimental data that were argued to support the path integration hypothesis (Behbahani et al., 2021; Kim and Dickinson, 2017) have not adequately excluded the possibility that the results may include a contribution from a pheromone-based mechanism.

### Displacement experiments provide evidence for path integration in *Drosophila*

Next, we asked whether flies can use path integration, independently of possible use of pheromones, to return to rewarding locations. To do so, we implemented a procedure in which flies walking in a circular arena in darkness were automatically rewarded when they entered a 5.6 cm diameter circular reward zone (RZ) via optogenetic stimulation of sugar-sensitive taste neurons with a computer-controlled LED. Following the reward period, we waited for them to walk by chance onto a thin slider and then moved them from the RZ vicinity – and thus away from any emitted chemicals or other sensory cues physically linked to this location – and displaced them by several centimeters (Fig. 2, details in Materials and Methods). If flies use path integration as a dominant mechanism to return to the reward location as previously proposed (e.g. Kim and Dickinson, 2017), they should return to the ‘fictive RZ’. This would be consistent with flies remembering the reward location (and not compensating for, or not detecting, the displacement). Alternatively, if flies used pheromones or other cues physically linked to the site of actual reward, they should not particularly visit the fictive RZ but rather the actual RZ. By displacing the flies from the actual RZ, we reduced the potential effect of pheromones and, if flies can use multiple mechanisms, sought to increase the relative importance of path integration mediated returns.

Prior to testing if displaced flies went to the fictive RZ, we first checked if optogenetic stimulation was indeed rewarding in this setup. As expected, rewarded flies did spend a substantial length of time walking in the RZ during optogenetic stimulation and immediately afterwards, prior to displacement. Control flies, which did not receive the optogenetic reward, did not spend substantial time there (Fig. 3A).

Based on previous work in *Drosophila* (Bell et al., 1985; Kim and Dickinson, 2017; Corfas et al., 2019), we focused on the observation that rewarded flies depart and return to the location of reward. In the 100 s test period, 13 of 19 rewarded flies visited the fictive RZ, compared with 1 of 20 control flies (two proportion *z*-test: z=4.13, *P*=3.7e^–5^). Thus, optogenetic reward led to a higher probability of visiting the fictive RZ after displacement. The actual RZ was also visited more frequently by the rewarded flies than by the non-rewarded flies (9/19 rewarded versus 1/20 non-rewarded; *z*=3.03, *P*=0.002). Next, we tested whether rewarded, but not control flies, tended to walk more in the vicinity of the fictive RZ after displacement. This would be consistent with them remembering this location and concentrating local search there. Histograms of walking frequency suggested that rewarded flies indeed spent substantially more time walking near the fictive RZ compared with the actual RZ, consistent with the path integration hypothesis (Fig. 3B; Fig. S3C). Notably, the peak of walking activity in the fictive RZ in the rewarded flies suggested that flies remember both the distance and direction to the rewarding location. Quantification shows that rewarded flies spent statistically significantly more time in the fictive RZ than the actual RZ, whereas control flies did not (paired *t*-test: rewarded, *P*=0.029; control, *P*=0.572; Fig. 3C).

If flies used path integration in these experiments, one might predict that their initial motion after displacement would be in the direction of the remembered reward; for example, if return to the reward was triggered by displacement. We thus examined the direction of movement during first 5 s after displacement. Rewarded flies moved on average in the direction of the fictive RZ after displacement (Rayleigh test for uniformity: *P*=0.018, z=3.894; mean angular deviation from the fictive RZ center: 28.9 deg), whereas control flies did not move in any consistent direction (Rayleigh test: *P*=0.352, *z*=1.057; mean direction relative to the fictive RZ center: −92.0 deg) (Fig. 3D,E; Fig. S3E).

Theoretically, the results so far are consistent with a model in which rewarded flies emit a pheromone trail along their path and, after displacement, return along this chemical trail on the surface of the displacement slider. We ranked the flies by the amount of intersection between their trajectories just before and just after displacement (Fig. S3F,G). Only four trajectories out of 39 had an intersection value of 1 cm or greater and where it looks like such route following may have occurred (Fig. S3F). Two such trajectories were from rewarded flies and two from non-rewarded flies. Additional quantification did not reveal differences between the rewarded and control groups (Fig. S3G). These results are inconsistent with the pheromone trail hypothesis, as are the results of a previous study that addressed a similar question (Brockmann et al., 2018).

## DISCUSSION

Using experiments and modeling, we showed that flies emit pheromones that could be used to guide the fly to return to a previously rewarded location. Such chemical cues may be a simple yet effective strategy to return to rewarding locations and our data suggest that this possibility must be carefully controlled in studies of path integration behavior. Taking this potential confound into account, we performed a displacement experiment on freely walking flies which tested the possibility that flies use path integration to return to a remembered location of previously experienced optogenetic reward. Our results support the hypothesis that flies are able to use path integration to maintain an estimate of distance and direction to the location of a prior reward. These results thus add to the growing evidence that *Drosophila* can perform path integration but suggest careful delineation of pheromone-mediated from memory-mediated mechanisms will be required as spatial navigation behaviors are further investigated.

Future studies could investigate the chemical composition of the deposits seen here and how they relate to deposits known from existing work (Abu et al., 2018; Duménil et al., 2016; Keesey et al., 2016; Lin et al., 2015). By storing information about the environment in pheromones, the ultimate effect – of returning to a previously rewarding location – may be achieved with a substantially simpler computational strategy.

In addition to path integration, the navigational capabilities of freely walking *Drosophila* include returning to rewarding locations using vision (Foucaud et al., 2010; Ofstad et al., 2011) and using a working memory for visual landmarks (Neuser et al., 2008). It will be interesting to study how path integration interacts with other navigational strategies and how additional sensory input, such as visual cues interact with the path integration abilities we studied here.

Several recent studies have taken advantage of *Drosophila* fixed rigidly to record neural activity in the central complex – a key neural substrate for navigation behaviors – during walking or flying behavior (Fisher et al., 2019; Giraldo et al., 2018; Green et al., 2017; Green and Maimon, 2018; Kim et al., 2017; Lu et al., 2022; Seelig and Jayaraman, 2015; Turner-Evans et al., 2017). Although restraining the animal facilitates brain imaging and electrophysiology, it may also limit behavioral performance as it changes biomechanics in addition to disrupting the multi-model input an animal would normally receive as it walks or flies freely. Preventing natural, free movement is known to disrupt normal visuo-motor coordination in flight (Stowers et al., 2017) and we predict that tethering also may alter navigational abilities. The evidence in support of path integration we presented here and that in the literature comes from experiments in freely walking flies (Behbahani et al., 2021; Brockmann et al., 2018; Corfas et al., 2019; Kim and Dickinson, 2017; Stern et al., 2019). One recent such study argued that intact PFNd neurons are required for path integration in a free-walking assay (Lu et al., 2022). An open question is thus whether path integration or other natural navigation behaviors can be replicated in a tethered behavioral apparatus.

More generally, efforts to bridge the gap between the ability to perform physiological recording from tethered flies and the ability to study behaviors of freely moving flies will be useful as we seek to investigate the circuits for navigation in *Drosophila*. Emerging and future work will seek to increase the realism of tethered experiments, for example, by increasing the sophistication of the feedback using virtual reality in tethered animals (Haberkern et al., 2019). Complementary efforts to perform calcium imaging of neuronal activity in freely walking flies attempt to bridge the gap from the other direction (Grover et al., 2020). A combination of neuronal silencing and activation in freely behaving animals in addition to physiological experiments in tethered animals also looks promising (Lu et al., 2022).

*Drosophila* may be an ideal system to investigate the relevant neural circuits and, ultimately, the biophysical implementation of path integration in one species of insect. Many species of insects are capable of path integration, with the list recently expanded to include *Scarabaeus galenus* dung beetle (Dacke et al., 2020). Path integration is a well-established component of the suite of navigational strategies used by Hymenoptera (Patel et al., 2022; Stone et al., 2017; Wehner and Wehner, 1986; Wittlinger et al., 2006) and has also been described in non-insect arthropods (Hemmi and Zeil, 2003; Hoffmann, 1983; Zeil, 1998; Zeil and Layne, 2002) and, recently, a fully aquatic *Neogonodactylus oerstedii* mantis shrimp (Patel and Cronin, 2020). Thus, the question arises as to whether the capability of performing path integration may have evolved in ancient ancestors of the Arthropoda and if the behavioral abilities and basic neural substrate may have been conserved since then. If so, *Drosophila* will serve as a useful model system across the insects and beyond.

## Supplementary Material

10.1242/jexbio.245289_sup1 Supplementary information
