# Supplementary material for: Displacement experiments provide evidence for path integration in Drosophila
Source: J Exp Biol. 2023 Jun 16;226(12):jeb245289. doi: 10.1242/jeb.245289 (PMC10281513; doi:10.1242/jeb.245289)
Supplement: Supplementary information [file jexbio-226-245289-s1.pdf]

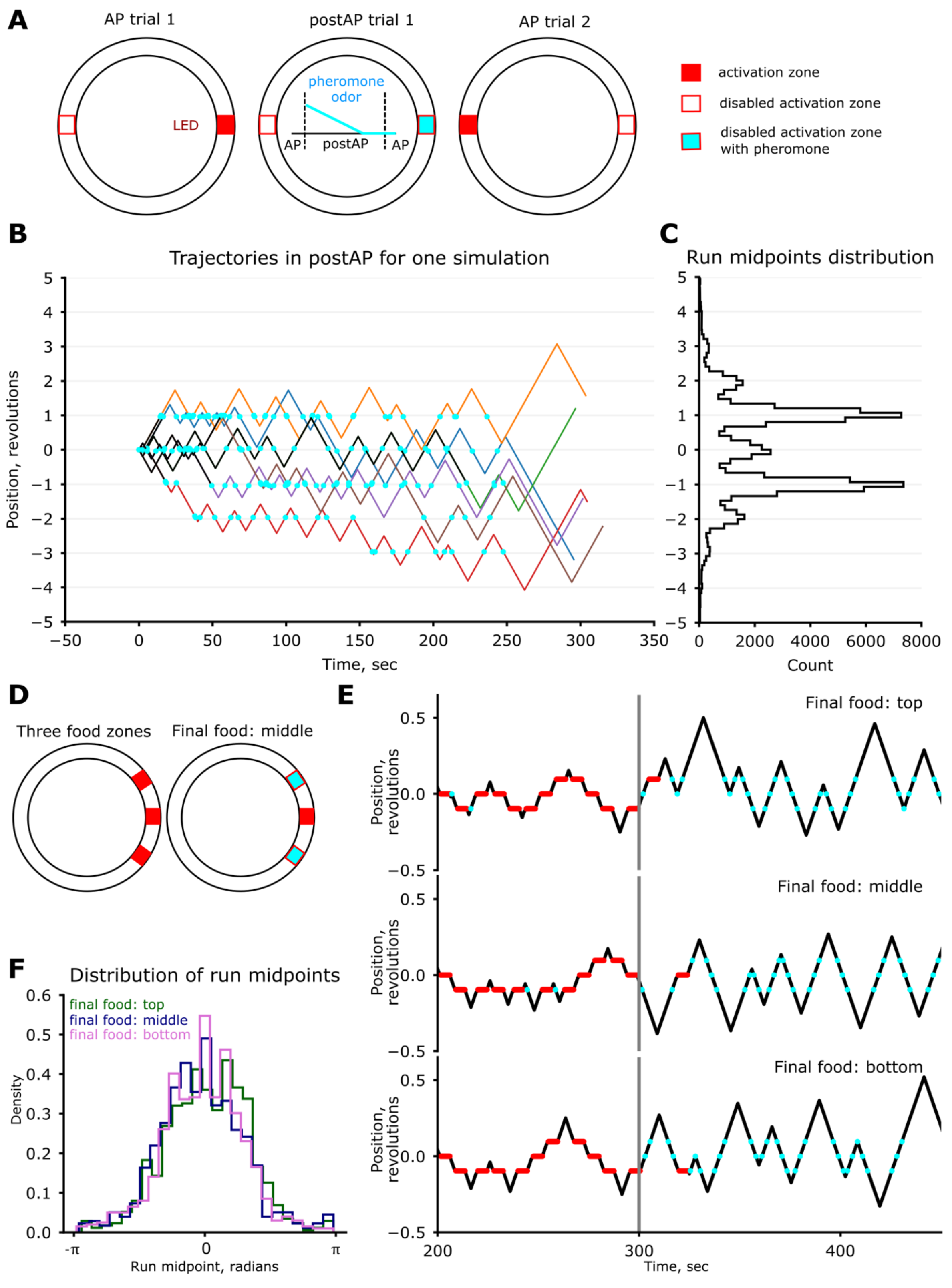

Journal of Experimental Biology • Supplementary information

Fig. S1. The pheromone model of Drosophila navigation in a circular channel. (A) Schematic of the small annular arena (26 body lengths circumference) with alternating activation zones. After being rewarded in an activation zone, the agent releases a pheromone with a decaying odor value (compare with Behbahani et al. 2021, Fig. 2). (B) Example pre-return (black) and post-return (colorful) trajectories of pheromone model simulation in a small arena. Time 0 corresponds to the last eating and is highlighted in red. The position is relative to the last reward. The moments where the agent feels the pheromone odor are highlighted in cyan. (C) The histogram of run midpoints of the post-return trajectories from 1000 simulations. (D) Schematic of the bigger annular arena (52 BL) with three food zones (compare with Behbahani et al. 2021, Fig. 6). For the last activation one of three food zones is used. The deactivated zones will have a pheromone signal. (E) Example trajectories of the pheromone model simulations for different final food locations. (F) The distribution of run midpoints after activation for different final food locations. Only trials where the agent visited all three food zones in last two AP runs were used. Total number of simulations: 1000 for each condition.

Journal of Experimental Biology • Supplementary information

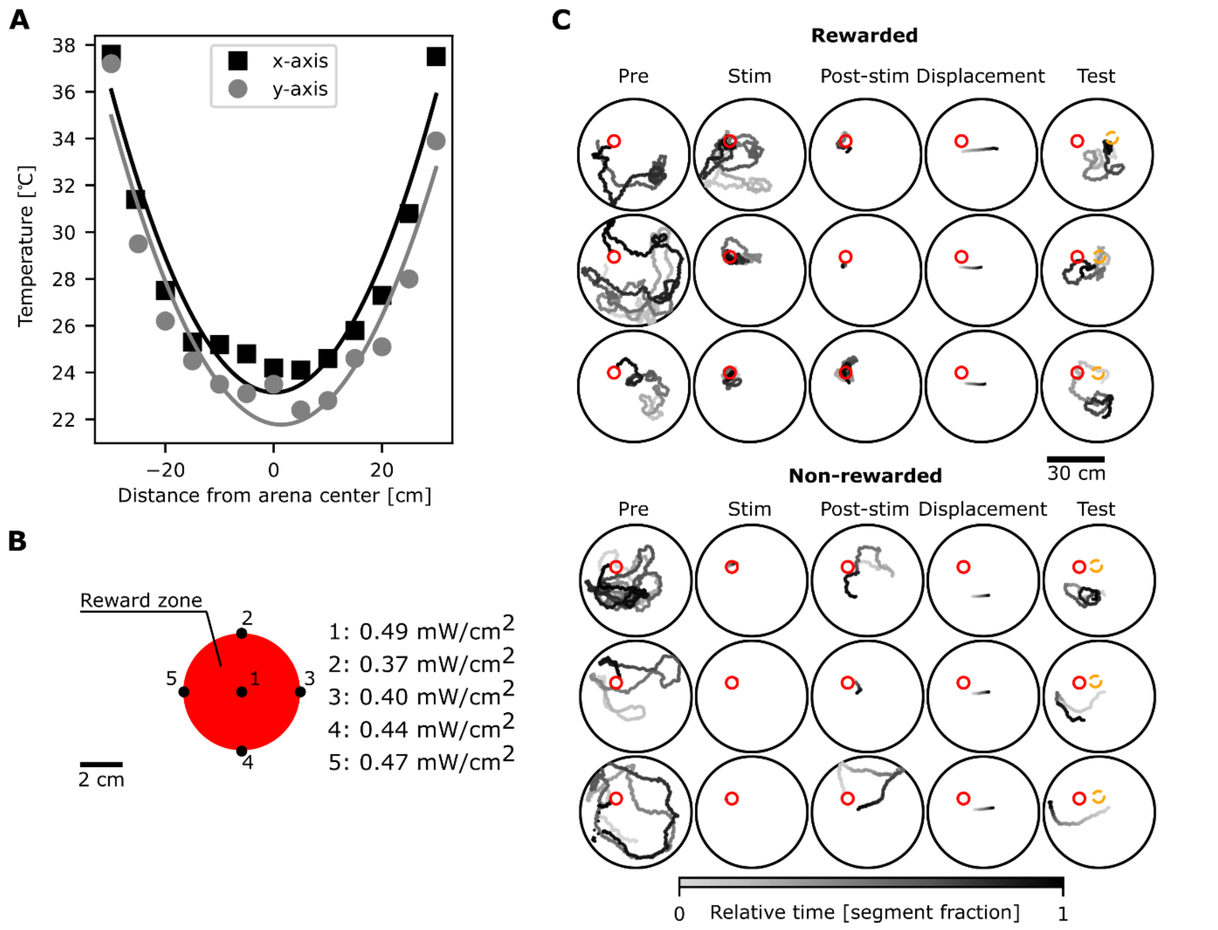

Fig. S2. Characterization of optogenetic stimulus. (A) Temperature profile along two axes with second degree polynomial approximations. (B) Values of stimulated light intensity in some points in the reward zone. (C) Trajectories of three rewarded and three non-rewarded flies during different experiment stages. Each row is from a single fly.

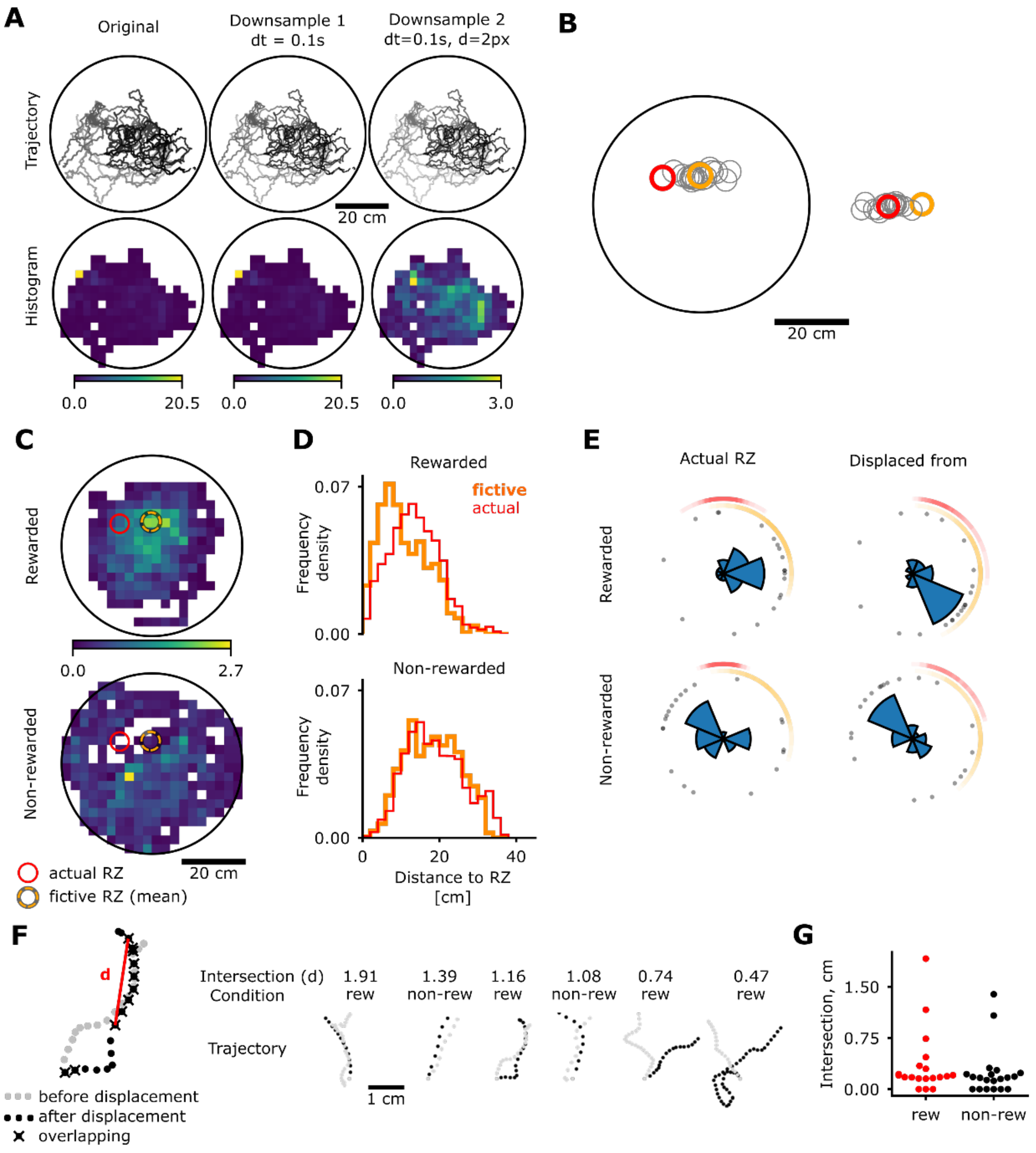

Fig. S3. Displacement experiment analysis. (A) Two stage downsampling to calculate walking locations from residency locations. Left to right: original --- frame rate 100 fps; stage 1 --- by time (step 0.1 s), stage 2 --- by distance (step 1 mm). (B) Coordinate transform: left --- original coordinate system, right --- fictive RZ coordinate system. Reward zones for all trajectories are indicated in gray, mean reward zones in color (actual: red, fictive: orange). (C) Walking frequency histograms during test period in the original coordinate system. Same data aligned to fictive RZ: Figure 3B. (D) Distributions of distances from all points visited by the flies during test period to fictive (orange) and

Journal of Experimental Biology • Supplementary information

actual (red) RZ. (E) Walking directions during first 5 seconds after displacement. Each dot represents one fly. The arcs show the range of directions towards the actual (red) and fictive (orange) reward zones. Compared to plots in Figure 3D, these are aligned wtih the actual RZ (left) and displacement direction (right) being vertically above the central position of each plot. (F) Left: Intersection score d – length of the longest overlapping segment of trajectory points before (gray) and after displacement (black). Included are points closer than 2cm to the displacement point. The trajectory after displacement is shifted back by the amount of displacement. Right: Trajectories immediately before (in gray) and after displacement (in black) with highest intersection scores. Rewarded flies: "rew", non-rewarded flies: "non-rew". (G) Amount of intersection between trajectories immediately before and after displacement, comparison between rewarded (n=19) and non-rewarded (n=20) flies. Each dot represents one fly. Mann-Whitney U rank test: $p = 0.230$.

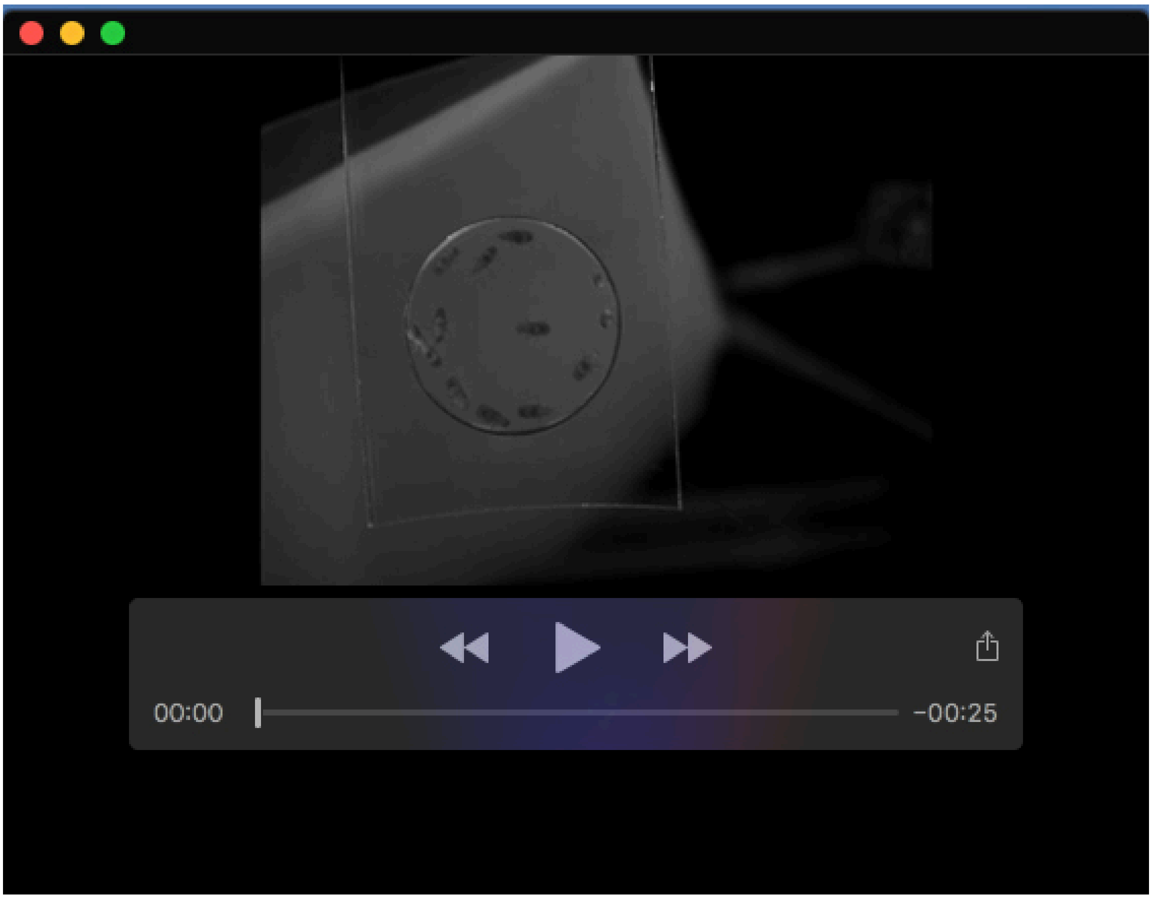

Movie 1. Chemical cues deposited by optogenetically rewarded flies. Video recording of experiment shown in Figure 1D.
